# Michael Rosentreter: Patientensicherheit lehren. Bedarfsanalyse und Konzeption eines integrierten Lehrprojekts für die medizinische Ausbildung

**DOI:** 10.3205/zma001152

**Published:** 2018-02-15

**Authors:** Christina Quandt

**Affiliations:** 1 Medizinische Hochschule Hannover, Klinik für Anästhesiologie und Intensivmedizin, Hannover, Germany

## Recension

The author Michael Rosentreter is a medical sociologist with many years of experience specialist nursing and has been researching and teaching patient safety since 2009, among other things as part of the research project “Error Reporting Systems for Patient Safety” at the Institute for History, Theory and Ethics of Medicine at the RWTH Aachen University.

Despite consensus in health care policy on the relevance of the topic "patient safety" and despite existing learning catalogs, a corresponding subject has not yet been established in the curricula of health professions and aspects of patient safety are touched at most selectively and more by chance. This book is intended as a practical guide to the argumentation for a compulsory subject “patient safety” and the planning of a teaching concept, approaching the subject but by for clinicians rather unfamiliar medical sociology and educational science side. The book is very textual, sometimes diffuse, practical and short clinical examples are almost completely omitted.

In a total of eight chapters, the author spans the arc from problem description, needs analysis, condition analysis and safety culture to curriculum analysis, justification of learning objectives and planning a teaching concept and thus completes the classic Kern cycle for curriculum development.

Initially, there is a strong emphasis on the still existing discrepancy between patient safety as a matter of few experts, transfer of patient safety in everyday clinical practice under multiple stressors and the patients expectation that, of course, medical treatment should not cause any harm. In order to bring about a real change of mentality in the discussion of errors, the author proposes to realize corresponding teaching projects early in the training of the health professions (not only of the medical!), preferably interdisciplinary and multi-professional.

With a conservative estimate of 18,800 deaths per year following treatment errors in Germany and a disproportionately low number of teaching programmes, training requirements are undisputed. In turn, the content of the training should cover higher-level competencies such as the formation of attitudes, thinking styles and awareness, although identification of learners need may be difficult because aspects of patient safety is hardly addressed in medical education.

The presented teaching concept of patient safety as a cross-sectional subject begins before first clinical assignments of medical students and continues with about half of the training period in the continuing medical education. The individual modules are described in great detail in terms of content and provided with method recommendations and are therefore actually useful as a practical guide. The modular structure depicts both the aspect of "lifelong learning" and hermeneutic learning. In my opinion, however, the author does not adequately discuss how the approx. 78 teaching units can be generated during Medical School and the 66-96 teaching units in postgraduate education. Also not answered is the question of mutual recognition when changing the place of study or the State Medical Association.

With more than 1,200 references and about 500 pages, "teaching patient safety" is more about "heavy food", which provides the expert with some well-known, but also numerous new aspects and further literature, but for the quick entry into the topic it is rather not suitable.

## Competing interests

The author declares that she has no competing interests.

